# Homo methodologicus and the origin of science and civilisation

**DOI:** 10.1016/j.heliyon.2023.e20237

**Published:** 2023-09-16

**Authors:** Alexander Krauss

**Affiliations:** a London School of Economics, UK; b Institute for Economic Analysis, Spanish National Research Council, Spain

**Keywords:** Origin of science, Foundations of science, History of science, History of scientific method, Origin of scientific method, Origin of civilisation

## Abstract

Few things have impacted our lives as much as science and technology, but how we developed science and civilisation is one of the most challenging questions that has not yet been well explained. Attempting to identify the central driver, leading scientists have highlighted the role of culture, cooperation and geography. They focus thus on *broad* factors that are important basic preconditions but that we cannot directly influence. To better address the question, this paper integrates evidence from evolutionary biology, cognitive science, methodology, archaeology and anthropology. The paper identifies 9 main preconditions necessary for contemporary science, which include 6 main preconditions for civilisation. Using a kind of quasi-experimental research design we observe that some cultures (experimental groups) met the preconditions while other cultures (control groups) did not. Among the preconditions, we explain how our mind's evolved methodological abilities (to observe, solve problems and experiment) have directly enabled acquiring knowledge about the world and collectively developing increasingly sophisticated methods (such as mathematics and more systematic experimentation) that have enabled science and civilisation. We have driven the major revolutions throughout our history – the palaeolithic technological and agricultural revolutions and later the so-called scientific, industrial and digital revolutions – by using our methodological abilities in new ways and developing new methods and tools, i.e. through *methodological revolutions*. Viewing our methods as the main mechanism through which we have *directly* developed scientific and technological knowledge, and thus science and civilisation, provides a new framework for understanding science and the history of science. Viewing humans as homo methodologicus, using an expanding methodological toolbox, provides a nuanced explanation of how we have been directly able to meet our needs, solve problems and develop vast bodies of technological and scientific knowledge. By better understanding the origin and foundations of science, we can better understand their limits and, most importantly, how to push those limits. We can do so especially by addressing the evolved cognitive constraints and biases we face and improving the methods we use.

## Introduction

One of the most foundational and oldest questions is how we developed science and civilisation. Scientists have attempted to address this question taking one of three ways. One way is studying broad factors related to the evolution of intelligence. Evolutionary biologist Kevin Laland and anthropologist Joseph Henrich stress how our cognitive capacity for culture has been the central driver [1,2]. Developmental psychologist Michael Tomasello similarly emphasises the importance of social cooperation and coordination [3,4]. A second way scientists have attempted to answer this question is studying broad factors related to geography. Physiologist and geographer Jared Diamond highlights the role of favourable ecological and agricultural conditions in making these achievements possible [5,6]. A third way researchers have attempted to tackle this question is studying broad factors at a particular point in history. Common explanations for the origin of science in particular revolve often around 17th century Europe and include the expansion of capitalism and increased economic wellbeing [7], Christian worldviews [8,9] and the printing press [10] that fostered the work of scholars like Kepler, Galileo and Newton.^1^ [[11], [12], [13], [14]] These three approaches focus mainly on *what broad factor* can explain the development of science or civilisation – which we will see are both highly interconnected. The most common explanations are culture and geography – both of which have played an important role but we cannot directly influence these broad factors. We however have not yet specifically explained *how we have been directly able to develop* science and civilisation. In particular, we do not yet have an integrated explanation of the particular set of preconditions needed for science and civilisation and – among the preconditions – the human mechanism of how we have been *directly* able to influence and develop these achievements.

Explaining how we created science and civilisation represents an enormous interdisciplinary challenge given their great complexity and it requires taking a more holistic and methods-focused approach. We require integrating evidence from across science that enables explaining our mind's evolved methodological abilities needed to develop new methods and thus reason, acquire knowledge and do science in new ways. This is the aim of this paper. Only with such an integrated approach can we see how the different pieces fit together and identify what underpins them. Only with such an approach can we address the challenge of explaining how we humans are even capable of reasoning about the world and developing the methods and knowledge needed to create science and civilisation – that is, explain how these methodological abilities of ours developed over time. It is about explaining what makes the work of scholars like Aristotle, Newton, Darwin and Einstein possible in the first place. And whether our early ancestors, over our evolutionary history, used the same methodological means as we do today to acquire knowledge about the world.

Here we adopt this methods-driven and interdisciplinary approach that combines evidence from evolutionary biology, cognitive science, methodology, archaeology and anthropology, and synthesises the existing research that to date has been isolated across these fields. Applying this approach, we first outline the set of necessary preconditions for science and civilisation. And, among the preconditions, we secondly explain how *our mind's evolved methodological abilities (to observe, solve problems and experiment) and the new and increasingly complex methods we develop using these abilities (systematic experimentation, geometry etc.) are the central driving force through which we have directly created scientific and technological knowledge*, and thus science and civilisation. These are the paper's two main objectives and contributions to the existing literature.

With this methods-driven explanation of science, we can see how our early ancestors systematically observed, solved problems and experimented to be able to develop new methods including tools that have helped us better understand and control our environment and biology. This enabled them to learn how to decrease illness by developing new plant-based medicine techniques, to increase food production by experimenting with new agricultural techniques, and to increase our limited capacity to remember and process what we observe by creating new systems of written language and numeracy. Our ancestors then learned how to engineer boats, bridges and later pyramids that require systematic measurement, experimentation and developing geometry and principles of engineering. And they eventually created complex systems of mathematics and astronomy.

The present account here outlines the origin of science and civilisation as a gradual cumulative process throughout history: the development of civilisation is characterised as meeting 6 main preconditions, with humans developing *biological*, *basic cognitive*, *social* and *complex cognitive abilities* that account for our methodological abilities and have been fostered by favourable *ecological* and *demographic conditions*. In turn, the development of contemporary science is characterised as meeting 9 main preconditions, including – in addition to these – *written language and mathematics*, *social and political stability*, and *using our methodological abilities in more systematic and controlled ways to develop new methods and tools* (see Table 1). Science and civilisation, while often viewed as disconnected from each other, share 6 of the same preconditions. Civilisation provides the basis that allows for the other 3 preconditions needed for developing contemporary science. We cannot thus separate the two. We cannot understand the origins of science without understanding the origins of civilisation, and vice versa. Science has thus only ever developed as part of civilisation – that is, within civilisation. For the development of both, we require using our evolved methodological abilities that enable acquiring cumulative methods and knowledge.

We can frame these nine preconditions triggering contemporary science as a kind of quasi-experimental research design. Around the 1500s, the first eight preconditions were met in different cultures that thus had these same conditions, such as Chinese, Arab, Italian, German and English cultures. In some of these cultures, scholars tested and used *more systematic and controlled observation, measurement and experimentation to develop and apply new methods and instruments* to study the world – the treatment (precondition 9) – that they cumulatively built on over the next few centuries and embodies contemporary science. This was the case for instance in Italian, German and English cultures – which can be viewed as an *experimental group*. In other cultures at the time that also met the same eight preconditions, we illustrate that they too tested and used more systematic and controlled observation, measurement and experimentation to develop and apply new methods and instruments (the treatment) but not to the same extent and later scholars did not collectively build on the methods and instruments. This was the case for instance in Chinese and Arab cultures – which can be viewed as a *control group* that could have developed contemporary science. It is thus this set of well-defined preconditions that enables allocating different cultures into experimental and control groups that met the same eight preconditions, and then to test the treatment. While the different cultures within the experimental and control groups and between them each varied in different ways, all these cultures *independently* achieved these eight preconditions and all the cultures in the experimental group also met precondition nine. This illustrates that despite inevitable differences within and across cultures and experimental groups, these are robust and necessary preconditions. Some features of the ninth precondition arose, to different degrees, in various cultures, including Arab and Chinese cultures. We will illustrate how the probability of developing contemporary science was 0 before these preconditions were in place, and the probability then increased rapidly as the 6th, 7th and 8th precondition were met. So why did contemporary science develop and expand in the 17th century? The key spark was the invention of new major methods and instruments like the first microscope and then telescope that were not developed for research purposes but triggered a surge in new discoveries made possible with those tools that created a new foundation for studying the world.

Taking a step back, throughout our species' history including contemporary science, we have relied on our evolved abilities to observe, solve problems and experiment that also include reasoning causally, abstracting, testing hypotheses and categorising. This has formed the foundation of scientific methodology and our ability to do science. Science is best defined by the methods we use to do science, with contemporary science characterised by developing and applying new methods and instruments that enable more systematic and controlled use of observation, measurement and experimentation. How science is defined here is grounded in our species' evolution and it is consistent with but thus goes beyond the common definition as the study of the ‘world through observation, experimentation, and the testing of theories’ [15]. Civilisation is defined as a highly-complex ‘stage of human social and cultural development and organization’. [16]. We focus in this paper largely on scientific methodology, with no clear distinction made between scientific and technological knowledge.

We provide here a new general theory – the methodological toolbox theory – that explains how we have directly developed science and civilisation. It explains how we have done so by using our evolved methodological abilities of the mind (our *universal* methodological toolbox) that have enabled collectively developing new and more sophisticated methods (our *adaptive* methodological toolbox) and thus acquiring vast knowledge in increasingly systematic ways over time. How we are able to develop methods, which are the main mechanism driving science and civilisation, is better explained, understood and advanced within this more holistic framework. The study of the foundations of science has traditionally been the terrain of philosophy and history of science which has mainly adopted conceptual and theoretical approaches [[17], [18], [19], [20], [21], [22], [23]].

After this introduction, we summarise the existing explanations of the origins of science and civilisation and then present a new holistic and methods-focused explanation (Section 2). We then illustrate, comparatively across time, species and human cultures, how evolved methodological abilities of the mind lay the remarkable foundation for doing science. We outline how these abilities have been used in increasingly systematic ways by smart animals (Section 3), early humans such as homo erectus and Neanderthals (4), homo sapiens up to 11,000 years ago (5-6), inhabitants of early civilizations (7), ancient Chinese and Greeks (8) and scholars in the 17th century and onwards (9). We thereby compare the different groups systematically and integrate the evidence to identify the necessary and sufficient preconditions for science and civilisation (10-11). The present account here illustrates how our species’ success is best explained by our unique human ability to develop a myriad of new and increasingly sophisticated methods to meet our needs, solve problems and create vast bodies of knowledge – giving rise to a new kind of species called here *homo methodologicus* (Section 12). Finally we illustrate how our methodological toolbox has become a new source of selective pressure that has shaped the evolution of our mind (Section 13). We conclude by outlining the need to reduce our evolved cognitive constraints and improve our methods of science.

## 2 The current leading explanations of the origin of science and civilisation (culture and geography), and the need for a holistic and methods-driven explanation

Scientists have proposed different explanations for what has driven science and civilisation. Physiologist and geographer Jared Diamond emphasises the importance of favourable geographic conditions [5,6]. He summarises his central thesis as ‘History followed different courses for different peoples because of differences among peoples’ environments' [5]. He argues that factors related to ‘the rise and spread of food production is […] what I believe to be the most important constellation of ultimate causes’ [5]. Evolutionary biologist Kevin Laland argues as his main thesis that ‘our species’ extraordinary accomplishments can be attributed to our uniquely potent capability for culture [which means] the extensive accumulation of shared, learned knowledge, and iterative improvements in technology over time’ [1]. He stresses that high levels of social learning through copying each other's behaviour have been essential for our species' great achievements. ^(ibid.)^ Developmental psychologist Michael Tomasello also highlights the role of culture and in particular cooperation. His principal thesis is that ‘virtually all of humans’ most remarkable achievements—from steam engines to higher mathematics—are based on the unique ways in which individuals are able to coordinate with one another cooperatively’ [3]. He asserts that our species' ‘most distinctive characteristic is its high degree (and new forms) of cooperation’ that reflects our shared intentionality and allows for complex language. ^(ibid.)^ Anthropologist Joseph Henrich makes his central thesis clear in the title of his book ‘The secret of our success: How culture is driving human evolution, domesticating our species, and making us smarter’ [2]. He argues that ‘The key to understanding how humans evolved and why we are so different from other animals is to recognise that we are a *cultural species*’. (ibid.) It appears according to these leading scientists that if favourable geographic conditions that support high-productivity agriculture and food production emerge [5,6] or if cooperation, shared intentionality, language and learning abilities emerge [[1], [2], [3], [4]], then science and civilisation may arise.

However, how we have been able to develop a world of hand axes, lunar calendars and agriculture, and eventually microscopes, steam engines and randomised controlled experiments cannot just be explained with the central driving factor of geography [5,6] or culture [[1], [2], [3], [4]]. Culture is viewed as an all-encompassing notion involving all ‘self-created cooperative structures such as conventions, norms, and institutions’ [3]. Such factors are important basic preconditions. But they do not directly help provide guidance on the specific steps including *methodological* approaches we must take to develop such scientific and technological knowledge. And they do not provide guidance on how we expand our bodies of knowledge. Tomasello thereby aims to identify the differences ‘that ultimately lead to humans’ unique forms of cultural coordination and transmission (and so to telescopes and parliaments)’. [3]. cf [1,2,4]. But there are vast worlds between cultural coordination and transmission (or geography) on one hand, and telescopes and parliaments on the other. The two operate at fundamentally different levels and with *no direct* connection between them. An explicit shift to our methodological abilities of the mind for observing, problem solving and experimenting and especially developing new and better methods, then and now, provides a new perspective that *directly* connects the vast worlds between early homo sapiens on one hand, and contemporary scientists and engineers on the other. For our species has always required using these same methodological abilities and approaches to be able to acquire knowledge about the world and develop new technologies, and we have done so in more systematic and collective ways over time (Sections 3-12). Existing accounts do not yet provide a direct mechanistic explanation that connects our early origins with the methods we use to reason, do science and acquire scientific and technological knowledge [[1], [2], [3], [4], [5], [6],^8^,^24^]. The methodological toolbox theory presented here provides such an integrated explanation. To explain science and civilisation we should not focus on a single cause for human success and uniqueness – for example shared intentionality [3,4] or agriculture [5,6] – but instead on a set of methodological abilities that enable observing, solving problems, experimenting and developing new methods like mathematics using these abilities collectively (our *universal* and *adaptive* methodological toolbox). New methodological approaches are also more directly observable and measurable in the technological tools, artefacts and knowledge we have developed using them, than are basic factors like culture, cooperation, collective intentionality, language and geography (ibid.), as illustrated in this paper.

We will show how we humans drove the palaeolithic technological revolution and (once favourable geographic conditions emerged) the agricultural revolution. We then brought about (once favourable demographic conditions also emerged) the civilisation revolution. We were *directly* able to drive each of these revolutions and then the so-called scientific, industrial and digital revolutions through new *methodological revolutions*. To connect basic factors like geography and culture to the rise of human technology, agriculture, civilisation or science – and especially to how we have developed them – we need to place our methodological abilities and newly developed methods and tools at the centre of focus. For it is only by developing new and innovative methodological approaches that we made these advancements possible. To be able to drive the palaeolithic technological revolution at least 50,000 years ago, early hunter-gatherers had to use our methodological abilities in new ways and develop new tools like bows and arrows. This required a preconceived idea of the tools before creating them (imagination), reasoning causally and experimenting to identify better ways to produce them (Sections 3-5). To be able to spark the agricultural revolution at least 12,000 years ago, early hunter-gatherers had to use our methodological abilities in new ways and develop new methodological techniques of experimenting with seeds and understanding the causal interactions between seeds, rain and soil fertility (Section 5). To be able to bring about the civilisation revolution and thus the first large societies at least 6000 years ago, early village dwellers had to develop new methods including systems of mathematics including geometry to create buildings and monetary systems by planning collectively, measuring systematically, experimenting and predicting outcomes (Section 7). To be able to give rise to the so-called scientific revolution around the 17th century, scholars observed, measured and experimented in more systematic and controlled ways to develop new methods and tools that enabled a new understanding of the world. Galileo for example discovered new astronomical objects using the new telescope [13] and Newton created a new foundation for physics using the newly developed methods of calculus (Section 9) [14]. To be able to start the industrial revolution and thus mechanise our means of production around the 19th century, engineers and entrepreneurs had to continually create new methods and techniques to produce iron, textile spinning machines and steam engines through greater experimentation, systematisation and at times understanding of the causal mechanisms at work. To be able to spur the digital revolution and thus digital technologies in the 20th century, scientists and specialised professionals had to collectively produce new and increasingly efficient transistors, computers and the internet that shape our modern society. This required developing new mathematical and computational methods and continual experimentation.

Using our mind's evolved methodological abilities and developing new methods and tools with them has been at the centre of each of these major revolutions. They have enabled us, as the main mechanism we *directly* influence, to develop new technologies, agriculture, civilisation and science. Expanding our methodological toolbox underpins all our major scientific and technological advancements. Future revolutions to our way of life, such as a possible artificial intelligence revolution, would also be driven by developing new computational methods. Complex artificial intelligence systems would require computer and data scientists and systems engineers to develop new and more complex deep learning programmes, data processing methods and computer technology. But how have we even been able to start science in the first place?

## 3 Science of smart animals

How did we humans, as biological organisms, come to develop such a useful methodological toolbox that enables understanding the world and doing science? In order to survive and meet basic needs, species like ours have to acquire knowledge about the types of edible and poisonous foods, about their ecological environment and about other animals. They have to recognise regularities in nature. Many animals possess the cognitive and social abilities required to use basic tools and methods by systematically observing, solving problems, using trial and error, testing hypotheses, and learning and sharing them [25,26].

Chimpanzees for example use different types of tools and methodological techniques for extractive foraging. They use stone and wood hammers to crack nuts and sticks to extend their reach and extract termites and honey. They use leaf sponges to collect water, stones to throw as weapons and levers for different tasks [3,4,[27], [28], [29]]. Using different tools to solve different problems, chimpanzees have a toolkit that they acquire through learning and experimenting. ^(ibid.)^ For chimpanzees to use such tools requires that they have a clear objective of the tool in mind, predict how the tool can enable them to achieve that objective and understand how the tool needs to be applied. This requires them to understand the interactions between the tool, their hands and the desired outcome. Many other animals also use tools, from crows to sea otters and octopuses, by manipulating objects for their purposes and making inferences [27].

Chimpanzees can also represent reality abstractly and generate and test hypotheses about the behaviour of others – for example when they deceive others. Many animals also communicate and use forms of language [3]. Experimental research shows that non-human primates also reason about objects, space, quantities and the mental states of others, and identify causal relationships [3,4,27,30]. Omnivorous mammals – from rats to humans – reason causally by perceiving patterns and basing their behaviour on them. They make the connection between food with a peculiar taste and gastrointestinal sickness afterwards. (ibid.)

Overall, smart non-human animals are able to reason, acquire knowledge and use tools. They possess features we use to do science that include *biological abilities* (such as vision and other senses), *basic cognitive abilities* (observing, solving problems and categorising) and *social abilities* (using language and learning). They thus fulfil 3 of the 9 main preconditions for contemporary science (Table 1). These evolved abilities have been at the centre of how we and other smart animals think and solve problems over millions of years and remain central to science today.

## 4 Science of early humans

At least 1.5–2 million years ago, early humans like homo erectus and Neanderthals created complex tools like hand axes [31,32]. Stone toolmakers needed to represent mentally, infer and predict. Making hand axes requires systematically observing, experimenting, reasoning causally, testing a hypothesis, imagining and planning in order to produce a clear preconceived object. cf [33]. Such tool-making involves going beyond basic trial and error [34]. Early humans were able, about 600,000–1 million years ago, to control fire and then develop sophisticated fire-hardened spears about 400,000 years ago [24,25]. This requires complex reasoning, making interconnected inferences and assessing hypotheses [2,35]. These are the methodological abilities commonly used in contemporary science.

Overall, early humans, like other smart non-human animals, possessed features of how we do science and met 3 of the 9 main preconditions for contemporary science – *biological, basic cognitive and social abilities* that enabled developing increasingly complex methods and tools (Table 1). Because we know early human species created such complex tools [25] and because for us to create them requires a systematic approach and refining the tools, we know they also used these methodological abilities. There is no empirically-grounded alternative to explain the foundations of how early humans reasoned and acquired knowledge other than these methodological abilities they required using to develop the new tools.

## 5 Homo sapiens science (until about 11,000 years ago)

We homo sapiens are the result of millions of years of evolution and gradually emerged as a unique species in Africa an estimated 250,000 to 300,000 years ago [3,4]. Our evolution has shaped the way we reason about the world. As a result, we are able to observe, solve problems, experiment, categorise, reason causally, test hypotheses and develop new methods. cf. [27,36] How we acquire knowledge – both today and in the Palaeolithic – is based on these evolved cognitive abilities.

With increasing social cooperation, our early ancestors also developed a more complex system of language. This enabled describing what we observe and how we solve problems and share methodological techniques, tools and knowledge acquired through learning and teaching [1,3,24,30]. The ability to imagine and use abstraction and analogies also developed in our early ancestors, as seen in cave paintings, sculptures and other symbolic art during the Palaeolithic [25,37]. Contemporary scientists use this essential ability. Darwin for example used a model of the branches of a tree to illustrate his theory of evolution, with all species being related and humans just on one branch [38]. Across science we use representational models to simplify complexity – from theories and experiments to statistical simulations and mathematical equations. Our abilities for greater cooperation, language, abstraction and imagination have developed symbiotically. They have built on each other through cumulative feedbacks that have made these abilities increasingly complex over time [1,3], together with our abilities for developing more complex methods.

The end of the last ice age an estimated 11,000 years ago led to conditions for more rapid cumulative culture and knowledge. For it increased the diversity of plants and animals [5,6]. Using our methodological abilities we developed techniques to domesticate animals which requires knowing about biological reproduction, the nutritional needs of animals and selective breeding techniques to foster particular traits. We were able to develop new agricultural techniques in different regions of the world. This enabled producing a more stable food supply and a surplus of labour that we could spend on other activities [5]. To be able to successfully cultivate crops requires understanding how seeds, rain, soil fertility and erosion causally interact and often knowledge of annual cycles. It requires experimenting comparatively with seeds to produce higher yields, and selective planting techniques and often irrigation methods. ^(ibid.)^ We increasingly used our complex cognitive abilities for logical, rule-based reasoning. Early farmers had extensive botanical, zoological and ecological knowledge and were able to manipulate their environment through agriculture and livestock – as hunter-gatherer groups commonly do today [2,3,24,39].

Our evolved abilities, which cumulatively allow for method- and tool-making, developed through continual dynamic feedbacks between our material, cognitive and social worlds. cf. [1,24] We create knowledge either using only our mind's methodological abilities or applying more complex methods and instruments that we can only develop using these abilities. Our methodological abilities thus always drive the knowledge we develop – while we do not always apply the tools that we create ex-post using these abilities. Our tools are products of our methodological abilities that we use to solve problems more effectively or better study the world. Our methodological abilities and methods interact through a cumulative bootstrapping process in which they extend each other, in increasingly complex ways.

Population density, specialisation (the division of labour) and methodological diversity increase together, with changes in one often affecting the others. What explains the differences in the pace at which we have accumulated knowledge over the last ten thousand years are not vast differences in the cognitive resources we are born with. It is the diversity of methodological tools and techniques we have developed that are supported by greater demographic growth [3].

Overall, through these developments three further preconditions for contemporary science and civilisation emerged, namely increasingly *complex cognitive abilities* (for logical, rule-based reasoning) and favourable *ecological* and *demographic conditions* (Table 1).

## 6 The vast foundation of science and civilisation was in place at least 11,000 years ago

Taking an integrated methods-focused perspective, we can see how the vast foundation for science and civilisation had already been laid during the Palaeolithic at least 11,000 years ago. We observe this foundation in six necessary abilities and factors:

• *Biological abilities*: vision, other senses and related physiological functions;
• *Basic cognitive abilities*: systematic observation, problem solving, experimenting (including trial and error), categorising, causal reasoning, testing hypotheses and abstraction;
• *Social abilities*: language, learning and teaching;
• *Complex cognitive abilities*: logical, rule-based reasoning to develop more complex methodological techniques;
• *Ecological factors*: the end of the last ice age and developing agriculture and diverse newly adapted tools;
• *Demographic and socioeconomic factors*: increased population density, greater division of specialisation and cumulative knowledge.

Our universal methodological toolbox is reflected in these four abilities that we humans all share. Our biological and basic cognitive abilities mainly emerged before the Palaeolithic. Our increasingly complex social and cognitive abilities mainly emerged during the Palaeolithic. Our favourable ecological and demographic factors arose at the end of the Palaeolithic.

Overall, all 6 main preconditions for civilisation, and 6 of the 9 main preconditions for contemporary science, were already in place at least 11,000 years ago (Table 1). Early homo sapiens have however been largely forgotten in the history of science, because they did not leave us behind written evidence of their systems of language and numeracy. With this vast foundation already existing, why did we not develop contemporary science then? We outline the remaining three preconditions in the next three sections.

## 7 Early civilization science

As small hunter-gatherer clans shifted to agricultural livelihoods and grew into larger communities and eventually cities [5,6] we became able to develop methodological techniques and knowledge at an unprecedented rate. New methods and tools changed most aspects of our lives: our food, the structures we lived in and medical and technological advancements we could make. Our highly social species became even more social [2,3,30]. We made large leaps towards science: we developed the earliest known systems of written language and mathematics around 6000 years ago [5]. Babylonian mathematics developed algebra, Pythagorean theorem and quadratic equations. Written language and mathematics marked a transformational shift that enabled using our methodological abilities increasingly systematically. These new systems make recording what we observe easier and help process and remember information, make mathematical calculations and build on our methodological techniques and knowledge. ^(ibid.)^

Geometry can be traced back to around 5000 years ago in Mesopotamia and Egypt. It involved principles of areas, lengths, angles and volumes that we used for surveying, agriculture, construction and astronomy [40]. Norte Chico civilization constructed vast pyramids at least 4500 years ago, requiring – both then and today – developing principles in engineering, architecture and geometry that are grounded in systematic measurement and experimentation. An Egyptian medical textbook from about 1600 BCE provides detailed cumulative experimental techniques of dealing with injuries, fractures, tumours and various surgeries and it applies the methods of examining, diagnosing, treating and prognosis [41].

In the Old Testament, Daniel (1: 12–13) [42] describes an experimental trial: ‘Test your servants for ten days. Give us nothing but vegetables to eat and water to drink. Then compare our appearance with that of the young men who eat the royal food [and drink wine], and treat your servants in accordance with what you see.’ Only by combining our cognitive abilities can we – then and now – test such a hypothesis and conceive the design of such controlled experimentation. We need to apply causal reasoning to test whether a potential cause (a vegetarian, water-based diet) has an effect on people's physical appearance by systematically comparing the outcomes between the two groups after 10 days, and then deriving inferences from the outcomes to modify people's diets in the future. We then combined controlled experimentation with randomisation and blinding in the 19th century which further reduces bias [43].

Overall, by applying our methodological abilities we created *systems of written language and mathematics* that are a further precondition to exponentially increase cumulative knowledge. Our ancestors in early civilisations fulfilled 7 of the 9 main preconditions necessary for contemporary science (Table 1). The ancient Chinese, Mayans and others developed agriculture, civilisations and mathematical systems *independently* across different regions of the world by using our methodological abilities and approaches in new ways – illustrating the robustness of these preconditions.

## 8 Ancient Chinese and Ancient Greek science

Scholars in ancient China studied a wider range of phenomena than in earlier civilisations, from astronomical events and the properties of animals to magnetism and sound. They did so with a logical view of how the world is broadly construed [44,45]. Using a pragmatic experimental approach, ancient Chinese developed, as the first or independently, many more advancements than ancient Greeks: including immunization techniques, magnetic compasses, negative numbers and the ‘Pascal’ triangle, astronomical observations of novae, seismographs, irrigation systems and quantitative cartography [[44], [45], [46]]. Because the Chinese created a complex system of astronomical records, including star catalogues and observations of eclipses and novae, our records today go back millennia and it contributes to the body of knowledge that makes up contemporary science [44,47]. Such advances allowed ancient Chinese to control and predict nature through technology [48]. For ancient Chinese to develop smallpox vaccines required – just as today – complex understanding of the causes and effects of infectious disease, their interactions and how to control them. Smallpox vaccines decreased the mortality rates of the disease by 20–30% [44]. Medicine, geology, alchemy/chemistry, geography, technology and engineering were bodies of knowledge supported by the state [47].

While many cultures developed knowledge in geometry, astronomy and physics, ancient Greeks converted these into more theoretical fields. Ancient Greeks also helped create formal logic, natural history, ethnography and formal philosophy. Archimedes developed principles for levers and buoyancy that underlie physics and engineering [49].

In 1021, Ibn Al-Haytham then criticised Aristotle for largely neglecting the method of induction [50]. Al-Haytham collected rich experimental evidence to develop elaborate theories of vision, light and colour. He also described and systematically used what has been called the scientific method, namely conducting controlled experiments to prove a hypothesis using verifiable procedures and applied a mathematical approach that enabled producing reproducible results [50].

Overall, we observe greater *social and political stability* over centuries and even millennia among ancient Chinese and Greeks who thus fulfilled 8 of the 9 main preconditions for contemporary science (Table 1). We also observe greater logical, rule-based reasoning, applied mainly empirically by ancient Chinese and increasingly theoretically by ancient Greeks. Only through systematic experimentation and stability over the next 1.5 millennia were the Chinese able to design mechanical clocks and engineer the chain drive and long iron-chain suspension bridges. It enabled them to develop optics, magnetism, acoustics and hydraulic engineering, the first official book of all medicinal drugs at the time with techniques for their use and their effects, and the comprehensive *Yongle Encyclopedia* to document their vast knowledge [44,51]. By the 15th century, the Chinese had created the world's most sophisticated science and engineering system. ^(ibid.)^ [46] To make such tools and methods, Chinese scholars did not necessarily rely on mythology or the supernatural – but on empirical observation, problem solving and experimentation.

As long as we have existed, we have observed, solved problems, experimented and developed methods and technologies. These methodological approaches are expressed in the work of Archimedes and Democritus, outlined in detail in the 11th century by Al-Haytham [50], and reiterated in the 13th century by Grosseteste [52], Roger Bacon [53], Aquinas [54], and later in the 17th century by Francis Bacon [17], Galileo [55], Newton [14] and others. The term the founder of the scientific method has been attributed to each of these scholars.

## 9 How did we develop contemporary science around the 17th century? Using our age-old abilities of observation, problem solving and experimentation more systematically to develop new methods and instruments

How contemporary science developed in 17th century Europe is viewed as one of the great unsolved questions in science [11,56]. Common explanations centre on Christian worldviews, the expansion of capitalism and economic wealth, the printing press and greater political and social liberties that fostered the work of scholars like Copernicus, Kepler, Galileo and Newton [7,8,10,11,46]. Christianity has been argued to foster science through a belief in laws of the universe, analogously to scientific laws [7,8]. But Christianity emerged over 1.5 millennia earlier and did not bring about greater scientific and technological advancements than the Chinese or Arab cultures did over the same period [44]. The importance of Protestant Christian values [9,11,57] is also not supported by the fact that the earliest of these European scholars, Copernicus and Galileo, were Catholics. The spread of capitalism and economic prosperity has freed up time and fostered individualism for more people to dedicate themselves to studying the world [7,11]. But this does not explain why they studied the world more systematically. Paper and printing disseminate new methods and knowledge [10]. cf. [57] But they had been developed and used in China for about two millennia and movable type printing was brought to Europe in the 13th century [58]. Greater social liberties in Europe did not impede Chinese scholars in developing many more scientific and technological advancements before Europeans up to the 15th century [11,44,46]. Leading researchers do not agree on the causes [11,56,57,59,60]. Some historians appear to define away the larger challenge by narrowing the definition of science – with most influential books focusing on the period around 17th century Europe, and not on our evolutionary history and our methodological abilities and approaches enabling us to do science [7,8,10,11]. cf. [46,56,57,[59], [60], [61], [62], [63]] Existing studies do not yet place our evolved methodological abilities and newly developed methods and tools at the centre of focus that have directly enabled developing knowledge in more systematic and controlled ways over time. ^(ibid.)^

In doing so, this account of methods-driven science explains how science became more systematic but did not emerge as an entirely unique entity in 17th century as we have always relied on our methodological abilities and approaches to be able to acquire knowledge. We have illustrated how the necessary *biological, basic cognitive, social and complex cognitive abilities* as well as *ecological and demographic factors* were in place at least 11 millennia ago, and they enabled doing science and developing civilisation; and the necessary *systems of written language and mathematics*, and *social and political stability* were then in place since the first civilisations and enabled acquiring vast bodies of knowledge (see Table 1). Scholars around the 17th century did not invent or directly influence our mind's evolved methodological abilities or our external conditions. What they did was build on that vast foundation of science laid before them. They used our universal methodological toolbox in increasingly systematic and controlled ways to develop a more sophisticated set of methods and instruments (our adaptive methodological toolbox). How we did science thus gradually changed through *more systematic and controlled observation, measurement and experimentation using new and more powerful methods and instruments* – as the key difference observed in the work of the pioneering scholars compared to earlier work. This was the remaining precondition and they thus fulfilled all 9 main preconditions: *contemporary science developed around the* 17th *century by using our methodological abilities to develop six revolutionary methods and instruments that enabled most discoveries at the time, namely using the newly developed microscope in 1590, telescope in 1608, barometer in 1643, air pump in 1659, statistics in 1663 and calculus in 1675*. Galileo, Hooke, Boyle, Newton and their contemporaries applied one or more of these methodological innovations to expand our understanding in astronomy, biology, physiology, pneumatics, mechanics and optics. These remarkably powerful new methods and tools mark the change from science to contemporary science as they enabled entirely new access and understanding of the world. These methodological innovations are the main mechanism driving this change. Our adaptive methodological toolbox developed over time to make contemporary science possible through:

• *New methods and instruments*: using our universal methodological toolbox more systematically to develop new and more complex methods and instruments that built on previous methods and instruments – which we observe in *all* major discoveries in the 17th century;
  o *Mathematical measurement*: using our universal methodological toolbox with more quantification that we already used to develop complex instruments but began applying also to better explain phenomena – which we observe in *some* major discoveries in the 17th century;
  o *Theoretical methodology*: using our universal methodological toolbox more explicitly that enabled a more theoretical understanding of methodology and science – which we observe in *some* major discoveries in the 17th century.

We can best understand scientific change at the time in light of this incremental methodological change – as greater systematisation in our methods. These features were already partly embedded in our means of producing technologies and in the works of earlier scholars that become more methodical over time.

While we used methods and instruments more systematically, the common explanations of geography and culture including religion did not fundamentally change. Such factors are not directly reflected in the work of these leading scholars and are construed too broadly to explain the particular methodological change that took place in the way they studied the world. To address the question we have to explain why these scholars observed, measured and experimented with more systematic methods and tools that could initiate a chain reaction among them and later scholars.

(i) More new methods and instruments

The key spark that spread science on a no-return path and captured the attention of the public and governments was the invention of the first major instruments used for science. So *why* did contemporary science develop around the 17th century? The first two major scientific instruments – the microscope and then telescope – were developed for non-scientific purposes, before most of the work of the major scholars of 17th-century science. The first microscope was invented in 1590 by Zacharias Janssen, a Dutch eyeglass maker, using a simple converging lens of glass beads [66]. This groundbreaking invention enabled us, for the first time, to vastly expand what we can observe in the world. It inspired wider interest in studying the world around us and led to the invention of the telescope in 1608 by Hans Lippershey, also a Dutch lensmaker. Janssen and Lippershey did not do scientific research and developed these two tools without research purposes in mind. But the tools were quickly applied to study the world. They led to the discoveries of capillaries, cells and bacteria, the moons of Jupiter, the motion of stars, and galaxies. The probability of making these discoveries was 0 before such tools were developed, and the probability then jumped when such tools were created. These new instruments enabled these major discoveries that were unknown phenomena to the inventors of these tools. *Because these tools were not developed with the intention of being used as scientific tools to make different breakthroughs, the tools at first lacked a relation to the outcome (the breakthroughs). Using quasi-experimental reasoning, this lack of an initial relation to the outcome is the key to identify the direct causal effect of the new tools in bringing about the breakthroughs that they were not even developed for.* Establishing that the independent variable (the new tool) had no initial relationship to the outcome (the new breakthrough) helps isolate the causal effect of the independent variable in driving the outcome and helps mitigate alternative explanations (so-called confounding variables). So we can establish here the cause (the new tools) and effect (the new breakthroughs). This illustrates that these powerful new tools are the causal mechanism of major discoveries of 17th-century science, without which they would not have been possible and with which they were largely inevitable. So while early scholars explored questions about the world, the first large-scale surge of interest in science that led to a growing group of scholars studying the world came with these breakthrough instruments. These two tools triggered the development of other successful tools such as the barometer, air pump, statistics and calculus in the 17th century. Together, these six revolutionary instruments provided, for the first time, in millennia *an entirely new foundation for studying the world in a more systematic way* that no longer relied heavily, or at all, on Ancient Greek knowledge as the principal source of inspiration. These tools divided earlier scholars from 17th-century scholars.

Taking a step back, in the centuries leading to the 1600s we already observed, measured and experimented more systematically across Europe to develop increasingly complex methods and technologies. These include magnetic compasses in the 12th century, mechanical clocks, windmills and eyeglasses in the 13th century and better quadrants in the 14th century. More sophisticated eyeglasses led to the microscope and telescope [67]. Cumulative technological advances in Europe began to surpass those in China around the 1600s [44]. Think of what we need to create these technologies, such as mechanical clocks in 13th century Europe – which had already been created in the 8th century in China [11,67]. We require a highly cumulative process of using systematic observation (to identify the best materials for constructing clocks), experimentation (to establish the best design methods), causal reasoning (to recognise the underlying causes and effects of the clock's parts) and hypothesis testing (to test how each piece fits together). We also require abstraction (to plan and imagine how to construct it), prediction (to foresee its use for time keeping), mathematical reasoning (to develop a system of precise units divided into 24 h and 60 min and seconds) and understanding (to establish the complex interactions of the parts). 17th century scholars (i) applied these same systematic methodological approaches used to create such sophisticated technologies, (ii) gained awareness and inspiration from such technologies and the methodological approaches used to develop them and (iii) often used the recently created technologies themselves – as observed in their works. Gilbert [65] and Kepler [12] were then able to develop theories of magnetism using magnetic compasses and the knowledge of ancient Chinese who created them and brought them to Europe [67]. Galileo was then able to discover the moons of Jupiter using the new telescope [13]. Developing a mechanical clock can be, in some ways, as scientific and requires as much complex reasoning, experimentation and prior knowledge as required for Galileo to later discover the moons of Jupiter by using a telescope or to establish that objects of different weights reach the ground simultaneously by dropping and testing them. With mechanical clocks, Kepler, Boyle and Leibniz began to think of nature as operating analogously to a mechanical machine [68,69]. New methodological and technological advancements could motivate these scholars and serve as a metaphor for studying the world more systematically [59].

These scholars increasingly tested and applied the successful methodological approaches underlying the new technologies for more types of questions. We gradually applied new methods and tools not only to questions whose practical relevance was immediately clear (technological knowledge) but increasingly to those not immediately clear (purely scientific knowledge). Developing technology and science go hand in hand, with each providing reinforcing feedbacks over time. New tools and technologies enable approaching old questions in new ways and open new questions – as the six new revolutionary methods and instruments in the 17th century did.

The development of science is the making of our mind's evolved methodological abilities and the new methods and instruments we develop using these abilities in increasingly controlled ways. These have had continual feedback effects on expanding how we study and understand the world. The result of these feedbacks is a cumulative process in which greater methodological and technological advancements, and greater awareness of how to develop them, have led to a runaway dynamic with reinforcing effects that has given way to our expanding bodies of knowledge.

(ii) Greater mathematical measurement

In centuries leading to the 1600s engineers, architects, surveyors, navigators and watchmakers increasingly used quantitative methods for their work, which they often combined with experimentation [7,57]. Using these successful quantitative approaches already applied in these practical professions, a small but growing group of scholars around the 1600s were able to make more theoretical scientific advances. Copernicus made the first mathematical calculations of planetary trajectories to confirm that the earth revolves around the sun [64]. Gilbert used systematic observation, measurement and experimentation to investigate magnetism and electricity [65]. Kepler measured his observations using mathematical calculations to develop his three laws of planetary motion [12]. Newton used mathematical methods to create a theory of gravity and developed calculus to create a new physics of (classical) mechanics [14]. These scholars used mathematical methods to quantify their observations.

Yet leading medical and biological discoveries of the 17th century, such as the circulation of blood in 1628 and capillaries in 1661, were made experimenting with the newly created microscope – not using mathematical methods [59]. How contemporary science arose cannot be reduced to mathematical advances. It is instead using our universal methodological toolbox with greater systematisation, control and at times measurement to develop a range of new methods and tools.

(iii) Greater theoretical methodology

Around the 17th century we began better combining one methodological approach (e.g. identifying causal relationships) with another (e.g. experimenting) to identify causal relationships using experiments. We began combining mathematical methods with experimentation to conduct more quantifiable experiments – with such methods observed in the works of Galileo [13], Newton [14] and their contemporaries. The notion of scientific methodology, in its explicit form, arose. A more methodological means of studying the world spread, namely that we, with more controlled methods, can better explain nature. The way we acquired knowledge more methodically became increasingly important relative to knowledge itself.

Al-Haytham [50], Grosseteste [52] and Roger Bacon [53] and then centuries later 17th century scholars again stressed greater unification of observation, experimentation and methods with theory. This generally provided for better explanations of causes, testable hypotheses and reproducible results. The methodological pieces needed for contemporary science thus already partly existed [53]. There is no reason why the incremental methodological change that 17th-century scholars undertook could not have taken place for example in the 11th or 13th century by building on the work and methods of those early scholars. The process was just not sufficiently cumulative among the individual scholars conducting frontier research at the time. The groundbreaking work and methods of Al-Haytham in the 11th century [50], without a small but growing community of successors (as Copernicus and Galileo later had), was not sufficiently built on by subsequent scholars. This includes Al-Haytham's experimental work criticising the geocentric model and his elaborate theory of optics, giving him the title of the father of optics and at times the father of the scientific method.

Newton's work is considered the highest achievement of 17th century science and had the greatest influence on physics for subsequent centuries [59,60]. In his most important book *Philosophiae Naturalis Principia Mathematica*, he systematically uses our cognitive abilities to observe, solve problems and experiment that evolved among early humans, and he expanded work on mechanics by Aristotle and Archimedes. He adopted the numerical system developed by ancient Indians and brought to Europe by Arabs. He used geometry developed by the ancient mathematician Euclid in Egypt. He expressed his ideas using Latin and on paper invented in China. He adopted the logical, rule-based understanding of nature of Ancient Greeks. He used algebra developed by cultures millennia before him and applied astronomical knowledge acquired by his predecessors [14]. This enabled Newton – and Leibniz – to create calculus that Newton applied to develop classical mechanics. Considerable knowledge and the methods needed to develop that knowledge were adopted by Europeans from our predecessors and absorbed into contemporary science. We stand on the shoulders of countless ancestors.

More generally, contemporary chemistry largely developed in the 18th century, biology in the 19th century, medicine in the 19th and 20th century, and psychology, microeconomics and sociology in the 20th century also by using our methodological abilities more systematically to develop new methods and tools. These include advanced statistical methods, controlled experimentation and x-ray methods. What enabled developing science was not just the focus on mathematics adopted by some 17th century scholars studying the physical world or just factors like capitalism or the printing press. Within this methods-driven framework, we can see how developments such as contemporary capitalism and the printing press were themselves made possible by applying our methodological abilities in new ways – greater measurement and systematisation in economic activities and used in means of production. Contemporary capitalism and the printing press, once developed, could then help reinforce science.

### Three models of the origins of science and scientific methodology

**Fig. 1:**
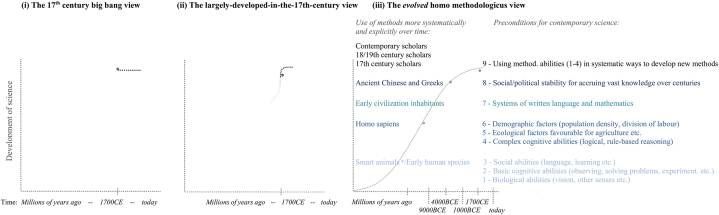
Three models of the origins of science and scientific methodology. The symbol * denotes using observation, problem solving, experimentation, causal reasoning etc.

How we developed science can thus be categorised into three views. (i) The most common view is that science, and scientific methodology, emerged in 17th century Europe [7,10,60] – what can be called the big bang view of science. For historians of science, the period around the 17th century ‘is the most important and talked-about era in the history of science’ [57]. cf. [60] (ii) A similar view is that science, and scientific methodology, emerged in 17th century Europe but has roots in ancient Greece. cf. [11,56,59] (iii) The holistic, interdisciplinary view outlined here is that science, and scientific methodology, developed as a gradual process evolving throughout our species' history. This reflects the use of our evolved methodological abilities more systematically to develop better methods over time – Fig. 1 (i-iii). All three views refer to science as the methods used to do science and a body of knowledge. ^(ibid.) cf.^ [15] All three views endorse the systematic use of observation, problem solving, experimentation, causal reasoning and testing hypotheses as the means to conduct research in contemporary science. The origin of science is viewed as commencing with Copernicus and Galileo (the first view) and including Aristoteles and Archimedes (the second view) because they are considered to be the first to use aspects of the so-called scientific method. Yet the third view outlines the evolutionary origins of these methodological approaches and how we have used them to different degrees throughout history. These approaches were then applied more systematically in the millennia and centuries before the 1600s to develop more complex methods and instruments, and especially following the 1700s and then 1800s and exponentially so today (Table 1).

Ultimately, to only view what European scholars have produced since around the 1600s as scientific would be a narrow interpretation of the history of science. Such a narrow view also neglects that science has been continually expanded since the 17th century. Developing computers since the second half of the 20th century has for example marked a further important advance in the way we do science since then. New computational methods have enabled doing science in ways that were not possible before, through big data and large-scale statistical methods, machine learning, artificial intelligence and internet. Computational methods greatly increase our productivity in systematically processing, analysing and sharing large sets of observations about phenomena in ways unimaginable in the 1600s. Applying computer technology marks a significant extension of our adaptive methodological toolbox and thus contemporary science. It has also helped lead to the digital revolution.

## 10 The preconditions for science and civilisation: a classification of the necessary methodological abilities and conditions

We have outlined the 9 main preconditions for contemporary science that include the 6 main preconditions for civilisation. We now provide a comparative framework that describes the similarities in these abilities and conditions across time, species and human cultures – Table 1. This framework illustrates that all homo sapiens – from early hunter-gatherers to Newton and Darwin – have always and will always use our evolved methodological abilities as the foundation for developing new methods and tools and acquiring knowledge. Smart animals and early humans meet preconditions 1–3, homo sapiens (up to 11,000 years ago) meet 1–6, inhabitants of early civilizations meet 1–7, ancient Greeks and ancient and imperial Chinese meet 1–8, and 17th century scholars meet 1–9, with contemporary science continuously expanding since then. There is nothing sudden about how this remarkable set of methodological abilities and conditions developed. The common approach in existing studies of focusing on a particular cause (e.g. culture or geography), particular time period (e.g. the 17th century), particular place (e.g. Europe) or a few particular scholars (e.g., Galileo and Newton) has held back our understanding of the origins and foundations of science. Yet scholars around the 17th century did make an important contribution: they used our already existing abilities in more systematic and controlled ways to develop or apply a set of new methods and instruments, with all other required abilities and conditions already laid (Table 1).

In summary, how then can we explain important shifts in how we became better able to acquire knowledge about the world? Ultimately, what distinguishes our species from other animals is our expanding methodological toolbox that enabled better combining our cognitive abilities and increasingly using logical, rule-based reasoning largely empirically. What distinguishes ancient Greeks from our earlier ancestors is our expanding methodological toolbox that enabled greater use of these abilities also theoretically. What distinguishes 17th century scholars from their predecessors is our expanding methodological toolbox that enabled using our abilities in increasingly systematic and controlled ways to develop more sophisticated methods and instruments. What marks the rise of contemporary science in the 17th century is the development of the six revolutionary methods and instruments that enabled the pioneering scholars at the time to make most discoveries. Our expanding methodological toolbox enables continually expanding science (Table 1).

**Table:**
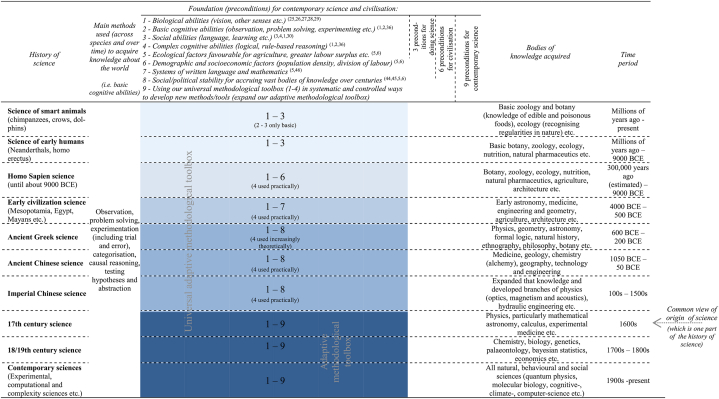
**Table 1**. The necessary preconditions for science and civilisation.

We should view the categories loosely and other features can be incorporated into these nine preconditions – such as other socioeconomic helping factors that are implicit in precondition 6 in contemporary society including scholars having access to basic health services, to academic conferences to exchange ideas, and the like. Other sciences – from Arab and Indian to American – were not included given the length of the paper.

Our evolved biological, basic cognitive and social abilities make our methodological abilities possible and are the three preconditions for being able to *do science*. We have thus had the ability to reason and do science for much of our species’ history. But that is not enough for developing contemporary science, which has nine preconditions (Table 1). In describing these preconditions, we observe that our methodological abilities including logical reasoning (preconditions 1–4) developed mainly beyond direct human control. Our favourable environmental conditions also emerged out of our control (5). We have however had an increasing, although limited, influence on how these abilities have evolved over the past hundred thousand years (discussed in the last section), while we have had a greater degree of control over demographic and social influences (6-8). Although we can do science without high population density and complex division of labour (6), without systems of written language and mathematics (7) and without social and political stability (8), science has not been and would not be sustainable without them. This is the reason why individuals, groups and societies throughout history who had the necessary abilities (1-4) and used them systematically to develop methods and acquire sophisticated knowledge (9) did not inevitably develop complex methods and vast bodies of knowledge that characterise contemporary science. These preconditions are so foundational for science that most people take them for granted and do not consider them scientific. This holistic and methods-focused explanation thus provides answers to the two biggest questions about the origins and foundations of science: what has directly enabled doing science and doing so more systematically? Meeting preconditions 1–3 and then increasingly precondition 4. And how have we expanded science over time? Meeting also preconditions 5–9. Science requires, in its systematic and sustainable form, all 9 preconditions.

None of these preconditions is contingent to one culture and not another. Just as civilisations developed in different parts of the world, so did science. Contemporary science could have developed in Chinese civilisation and almost did in Arab civilisation, where some features of all nine preconditions arose, with the work and methods of scholars like Al-Haytham. What if science's current methods and knowledge were to be lost through the decline of civilisation (as has occurred in the past)? To develop science from scratch in the future, we would not have to wait for a particular Western religious worldview to emerge [8], a particular capitalistic model to arise [7] or the like. *What would be required is fulfilling this set of nine general preconditions*. These nine preconditions can thus be used to predict how science can develop in the future.

**Fig. 2:**
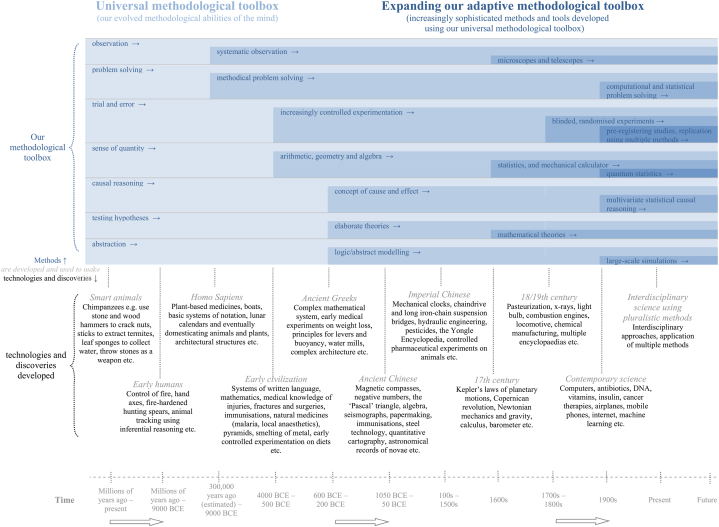
Evolution of more complex methods and tools, developed using our methodological abilities.

In Fig. 2 we illustrate the cumulative nature of our methodological abilities and increasingly complex methods and thus technologies and discoveries we have developed by applying them – in short, through cumulative *methodological revolutions*.

## 11 Thinking about how contemporary science developed – as a kind of quasi-experimental research design

We can frame these nine preconditions of contemporary science using quasi-experimental reasoning. The first eight preconditions were met around the 1500s in different cultures that thus had these same conditions, such as Chinese, Arab, Italian, German and English cultures. In some of these cultures, scholars tested and used *more systematic and controlled observation, measurement and experimentation to develop and apply new external methods and instruments* to study the world – the treatment (precondition 9) – that they cumulatively built on over the following centuries and embodies contemporary science. This was the case for instance in Italian, German and English cultures – which can be viewed as an *experimental group*. In other cultures at the time that also met the same eight preconditions they too tested and used more systematic and controlled observation, measurement and experimentation to develop and apply new methods and instruments (the treatment) but not to the same extent and later scholars did not collectively build on the methods and instruments. This was the case for instance in Chinese and Arab cultures – which can be viewed as a *control group* that could have developed contemporary science. These well-defined preconditions enable assigning different cultures into experimental and control groups that met the same eight preconditions (and developed vast bodies of knowledge and technologies), and then to test the treatment (the final precondition). *While the different cultures within the experimental and control groups and between them each varied in different ways, all these cultures independently met these eight preconditions and all the cultures in the experimental group also met precondition nine. This illustrates that despite inevitable differences within and across cultures and experimental groups, these are stable and reliable preconditions.* Some features of the ninth precondition also arose, to different degrees, in Arab and Chinese cultures. Many other cultures did not achieve the eight preconditions (with no systems of written language or mathematics or political stability) and thus did not develop contemporary science. How contemporary science developed and grew rapidly in 17th century Europe, not in Arab and Chinese cultures, was thus through the key trigger of creating new major methods and instruments in Europe, particularly the first microscope in 1590 designed for non-scientific purposes and with no intention for scientific discoveries. After the first microscope came the first telescope in 1608, constructed also for non-scientific purposes and without discoveries in mind. Once again, this lack of an initial relationship to making discoveries is the key to identify the direct causal effect of these new tools in still sparking a chain of new discoveries and contemporary science that they were not created for.

**Fig. 3:**
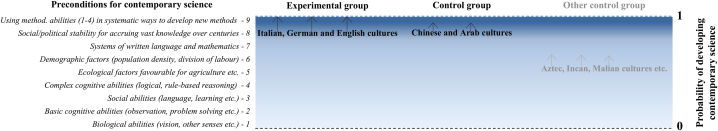
A causal framework of how contemporary science developed.

Importantly, the first eight preconditions did not emerge or were not designed in different cultures specifically with contemporary science in mind, but they still led to contemporary science. The probability of developing contemporary science was 0 before these preconditions were in place, and the probability then increased rapidly as the 6th, 7th and 8th precondition were met, as highlighted in Fig. 3. *These are necessary and sufficient preconditions for civilisation* *(1-6)* *and contemporary science* *(1-9)* *through the key factor of using our evolved methodological abilities in systematic and controlled ways to develop new methods and instruments – namely applying our universal and adaptive methodological toolbox.* This underlines the causal nature of these preconditions bringing about contemporary science. With the development of instruments like the microscope and telescope at the turn *o*f the 17th century, which happened to be created in Europe, came the first large-scale surge of interest in science that led to a runaway dynamic that expanded and consolidated science among a growing scholarly community and in society. Today, contemporary science is carried out in each culture that meets the nine preconditions that, as highlighted, can be used to predict science in the future.

What then is the main explanation and causal factor that we can directly influence – once the basic conditions were in place – for the difference between the way we acquired knowledge throughout human history and the way we acquired increasingly vast amounts of knowledge since the 17th century? Can the answer be as simple as the new methods and instruments we developed? Can developing the microscope, telescope, barometer, air pump, statistics and calculus around the 17th century, and then randomised controlled experimentation, electron microscopes, computers, big data methods and the like in subsequent centuries largely explain the accelerating pace of new discoveries? The response is yes when observing the major discoveries made in the 17th and subsequent centuries. The greatest legacy of Galileo, Hooke, Boyle, Newton and their contemporaries was not any individual discovery. It was developing and applying new methods for doing science, it was expanding the way we make discoveries.

## 12 Homo methodologicus: Our unique evolved ability of developing diverse methods to solve problems, acquire knowledge and do science

**Appendix Fig. 1:**
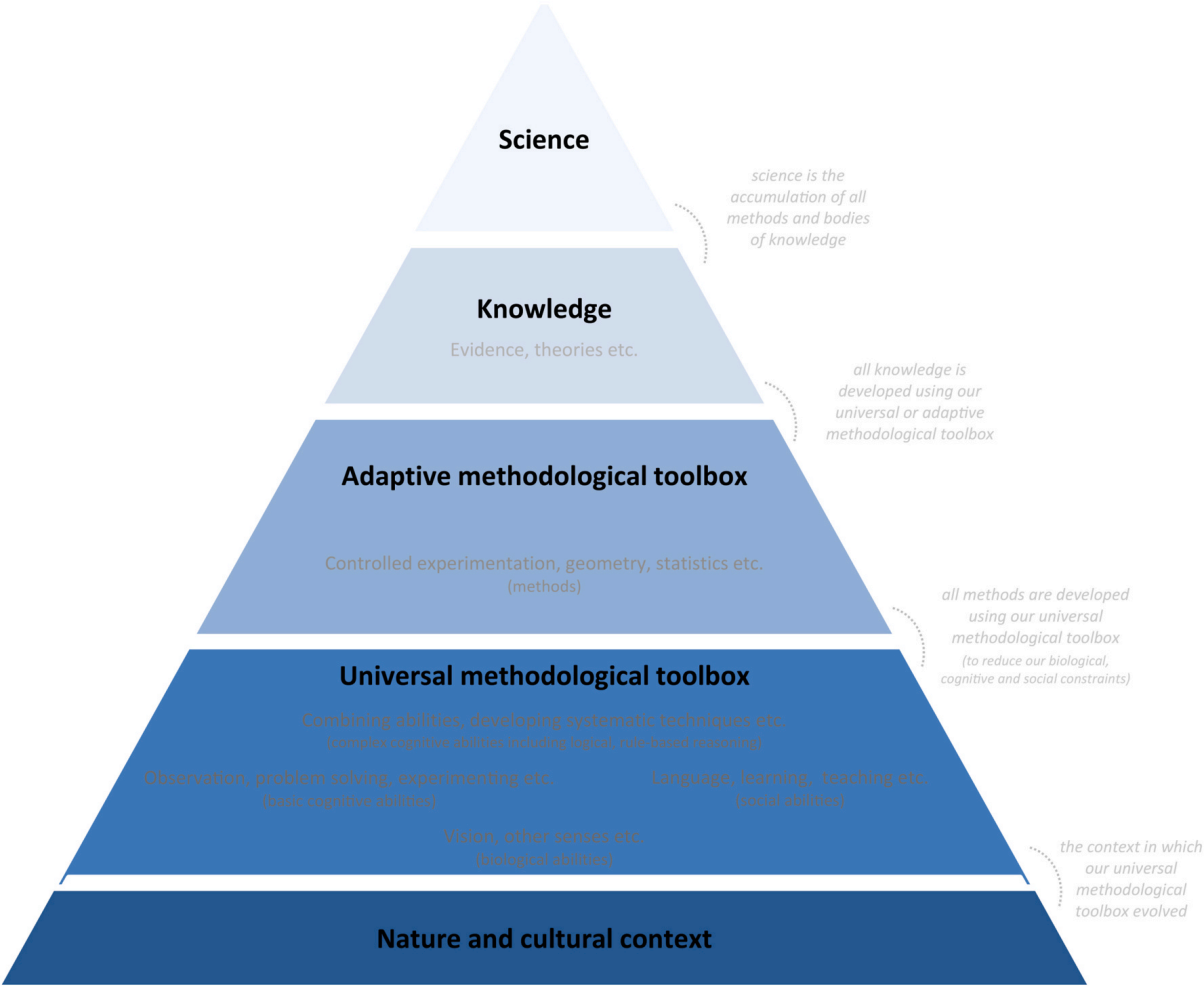
Methods-drive-science pyramid: The methodological foundations of science – how we use our methodological toolbox to develop knowledge and science

Our species’ extraordinary abilities to make sophisticated methods is the product of complex evolutionary feedbacks between nature and our biology, mind and culture. This evolutionary path we have taken has enabled our mind to acquire complex knowledge by developing increasingly complex methods. While other smart animals also have a sense of quantity, test hypotheses, communicate and use tools [3,4,27,30], only our species can perform statistical reasoning and cost-benefit analysis using these abilities. Being able to develop a diverse set of methods for solving problems distinguishes us, more than other factors, from other species: our species is uniquely a method-making species – what can be called *homo methodologicus* (Appendix Fig. 1).

Yet where does the mind end and the methods we create begin? We use our mind's internal abilities to develop complex external methods, and must interact with our natural and social environment to do so. Methods are, once created, external material artefacts in the world that can be shared and used by others – such as statistical and computational programmes.

Since our mind and senses have evolved and adapted to our environmental and cultural niche, we face constraints in how we make sense of the world especially beyond our niche. *Yet when we design a new method or instrument, we are asking how we can address a problem by reducing a human constraint we face in understanding the world. We are asking how we can answer a question by improving our existing cognitive or methodological abilities* (Fig. 2). New methods and tools we create expand our cognitive abilities – with mathematics for example enabling us to go beyond our basic sense of quantity.

We have used methodological diversity as a strategy to meet our needs and tackle challenges more than any other species. As our biology, mind and senses several hundred or even thousand years ago likely did not differ substantially from today, how we expanded our vast bodies of knowledge since then has mainly been through the more diverse methods we develop and share collectively. We are thus in part evolved methodologists and in part trained methodologists. The evolved methodologist in each of us has been an inevitable part of our cognition throughout our species’ existence. Every child is born with methodological abilities to observe, solve problems, do trial and error, and quantify. The *trained methodologist* in each of us has become an inevitable part of our cognition in our more recent history. Every child is trained in a set of methodological approaches and practices that commonly include arithmetic and geometry.

### 12.1 Our species’ most unique capacity: systematically using our evolved abilities to develop diverse and adaptive methods

Existing accounts of our species' uniqueness and success generally provide a single cause – such as shared intentionality [3, 4] or developing agriculture [5,6]. The present account of our *universal and adaptive methodological toolbox* outlines instead a set of evolved methodological abilities to develop more complex methods by applying these abilities collectively. Because it is the foundation for acquiring sophisticated knowledge it is arguably the most distinct feature of human life. While we are not the only tool-using species that communicates and cooperates [[1], [2], [3], [4]], we are the only complex method-making species. Only our species can apply the shared ability for trial and error to develop controlled experimentation, or go from the shared sense of quantity to develop arithmetic. Ultimately it is our ability to develop these adaptive methods using our mind's methodological abilities that has been the key missing link enabling us to revolutionise our understanding of the world in ways previously unimagined. This ability characterises our unique mind and endows us with a different relation to the world than any other animal. Our methodological toolbox set our species on a new methodologically-driven evolutionary course that had not yet been taken: making a new kind of methodological animal. Our ability to develop an adaptive methodological toolbox represents arguably the most unique feature in understanding the gap between the evolution of the mind of humans and smart non-human animals that is not just likely or can be imagined, but was necessary.

### 12.2 The closest we can get to a unified methodology of science: our universal methodological toolbox

Scholars have searched for a unified methodology of science for over a millennium [17,50,52,53,[70], [71], [72], [73]]. The nearest we can get to a unified scientific methodology – a core of science that we all rely on to acquire knowledge – is our universal methodological abilities to observe, solve problems and experiment. This explanation grounded in our evolution provides a natural foundation of science. Observation is thereby fundamental to nearly all knowledge we acquire. Our mind's methodological abilities are inherited regularities that link our human nature to science. This *methodological toolbox theory* presented here is as close as we may get to a universal theory of scientific methodology and the nature of science. For we, in our everyday lives and in science, have no choice but to use these evolved abilities or directly build on them. All human reasoning and all our views about the world rest on them. Our methodological abilities can resemble general purpose technologies, as they also serve as a foundation for a wide range of applications and innovations and can drive advancements across domains [74]. Yet while GPTs are commonly thought of as physical technologies (like computers or engines), methodological abilities and methods extend the notion to cognitive tools and approaches.

The central argument here: *viewing humans as homo methodologicus, using a universal and adaptive methodological toolbox, provides a more nuanced explanation of how we have been directly able to meet our needs, solve problems and develop technological and scientific knowledge, and thus science and civilisation. It provides a more nuanced explanation than commonly held views centred on geography, culture, religion or the like as the driving force behind these great human achievements*.

## 13 On the origin of science: our methodological abilities of the mind as a central mechanism driving human evolution

What mechanism has driven the evolution of our human abilities to enable us to develop technological and scientific knowledge? Scientists have proposed three main explanations for how we humans have evolved our intelligence to be able to reason in complex ways. One hypothesis is the *general intelligence hypothesis*. Significant differences evolve over time in intelligence, as a single factor, between humans and non-human apes that has been favoured by selection, with humans possessing greater processing capacity and memory and brains that are about three times the size [30,75]. A second hypothesis is the *adapted or ecological intelligence hypothesis*. Cognitive abilities, more broadly, mainly evolve due to adaptive environmental pressures, as observed for example in certain birds acquiring extraordinary eyesight for high altitudes and an ability to use magnetic fields to orient themselves and perceive their altitude [[76], [77], [78]]. In this sense, Darwin [79] and Wallace [80] argued that selection favoured individuals best able to adapt to their natural environment. A third hypothesis is the cultural intelligence hypothesis. Social-cognitive abilities evolve mainly as a result of challenges we face related to complex social interactions and cooperation [27,81]. These abilities have allowed us to develop distinct cultural norms and practices observed for example in complex language that have been favoured by selection [30]. In this sense, culture drives our evolution and thus our ability to develop science and civilisation [[1], [2], [3], [4]].

These cognitive, environmental and cultural hypotheses do not however outline the main mechanism that has directly enabled us to reason, better solve problems and develop increasingly sophisticated methods, technologies and knowledge. The three hypotheses are often viewed in contrast to or in parallel rather than complementing each other. An integrated approach has not yet been taken that combines these existing views and places the role of our methodological abilities of the mind – to observe, solve problems and experiment – at the centre of focus in studying human evolution and how these abilities have evolved enabling us to develop increasingly complex knowledge [1,3,27,30,75,[77], [78], [79], [80], [81]]. cf. [2,5,6,24] Here we provide a novel methods-focused explanation of how the methodological abilities of our *cognition evolved adapting to our natural and cultural environment* and have enabled us to reason, do science and acquire knowledge about the world in increasingly systematic ways over time.

This *methodological toolbox hypothesis* here explains how evolutionary selection would have favoured those members and groups, over our species' history, better able to apply our mind's methodological abilities in more systematic and successful ways. It would have favoured those better able to develop new useful tools like hand axes and spears and methods like lunar calendars and navigational techniques using stars. The members and groups of our species who have been more likely to meet their needs and survive are those who have been better able, especially over the past few hundred thousand years, to apply these abilities and develop increasingly adaptive methods and tools. These include creating plant-based medicines, using our imagination to design new technologies and eventually developing agricultural techniques and numerical systems to more effectively plan food production. Such successful methods and tools improved the fitness of those who developed and used them. This is how methodologically induced selection has led to our evolutionary development throughout human history. As our species, over the past hundred thousand years, migrated across the globe into new and unknown ecological environments, we were forced to adapt and readapt our methods and tools through a continual process of development and refinement. The result has been an adaptive methodological mind. So how would selection have taken place to support the preconditions for developing increasingly complex technological and scientific knowledge over time, and eventually science and civilisation? The members and groups of our species who would have more likely been selected are those better at applying our evolved methodological abilities – which have been fostered by favourable ecological and demographic conditions – including those who eventually achieved in early civilisations written language and mathematics, social and political stability, and learned to develop better methods and tools over time. This *methodological toolbox hypothesis* explains how our methodological abilities have, through continual feedbacks over time, functioned as one of the mechanisms of human evolution and main mechanism that we have increasingly been able to influence over time.

One of the great mysteries of our mind, human evolution and science – for cognitive psychologists like Steven Pinker [82] – has been the question: how have we, given our evolutionary history, evolved the ability to do science and develop highly elaborate knowledge that is not directly relevant for meeting our basic needs? How have we created calculus and surrealist art that are not directly needed for our survival? We have evolved abilities to reason logically that is required to create lunar calendars, plan for food cycles and develop numeracy and evolved abilities to use abstraction that is required to develop stone tools and shelters. Our evolved methodological abilities have thus directly helped us meet our basic needs and make sense of the world around us, as they do today. Within this methodological framework, we can explain how we develop logical methods (like calculus) and abstract objects (like surrealist art) by using these same evolved abilities for logical reasoning and abstraction but for purposes that do not directly influence our survival as when they first developed. This methodological toolbox hypothesis thus offers an answer to this mystery.

Environmental and biological factors impose selection pressures on all species and they governed our evolution for much of human history [1,3,24]. Yet over time we have learned to develop a larger set of new methods and tools to be able to better understand and control the environment and our biology in our favour. This has enabled us to increase our food production and reduce illness. Our abilities to develop and share innovative methods and tools across generations and build on them have become increasingly important and often exerted greater selection pressure than factors like the environment, animals, plants or disease in shaping our cognitive evolution. This is because the better the set of methods and tools we have created, the better we have been able to address and adapt to those external factors that change over time. What has shaped the evolution of our species and particularly our mind thus shifted from the largely external environmental challenges we faced over much of our past to the largely internal cognitive and social feedbacks. These have led to increasingly sophisticated abilities for method- and tool-making, in a symbiotic relationship together with greater imagination, cooperation and language, over the past few hundred thousand years. The methods and tools we developed using our mind turned into new selective pressures for our brain's evolution. With selection favouring those with better technology- and method-making abilities, our cognitive evolution would have been fostered by technological and methodological evolution in reinforcing cycles over time.

Methodologically induced selection can take place at the individual or population level. Our expanding methodological toolbox has been a driving force for demographic growth among different groups over history. Individuals and groups who were able to develop or acquire from others the set of experimental methods needed for agriculture and more efficiently exploit their environment were more likely to outgrow and outlive their hunter-gatherer counterparts. Some were then able to increasingly develop novel methodological techniques – from domesticating and breeding plants and animals, to irrigation methods and fertilisers. ^cf^ [1]. These enabled more stable surpluses of food and labour. Population and survival rates rose at a pace not yet experienced in human history – as documented in early cities and civilisations across the world [5].

With the development of agriculture, sedentary lifestyles and then early civilisations came a methodological revolution. More and more members of growing populations could dedicate their time to developing increasingly successful methods and tools. *Human populations throughout history that have rapidly increased their ability to survive and meet their needs have generally done so by expanding their methodological toolbox and so their ability to better understand and control the world around us*. Methodological innovation has driven our species' success. (The thrill and eureka-like feeling when making a new tool or discovery can be nature's way of saying that solving complex problems is important to us.)

Our methodological abilities and the new methods and tools we develop are more than just a result of our cognitive abilities. They are not just an outcome of our cognitive evolution. Our methodological toolbox is an essential part of explaining the interconnected evolutionary processes that in fact made our unique species and its mind possible – and our ability to develop science and civilisation. *Our methodological toolbox is both an inseparable product and driving force of evolution through an iterative process over time*. The evolution of our species’ mind is, more than any other species, a methodological and technological outcome of our own making – the making of our methodological abilities. We increasingly depend, with each newly adopted method and tool, on being taught how to use them and teaching them to others, from arithmetic to systematic experimentation.

Ultimately, this *methodological toolbox theory* explains how we have directly developed scientific and technological knowledge, and thus science and civilisation, by evolving and applying our mind's methodological abilities and new methods we create using these abilities collectively in increasingly sophisticated ways over time. It explains how we learned to solve differential equations, create experimental controls and tackle complex challenges that no other species can.

## 14 Conclusion

One of the oldest and most challenging questions is what the origins of science and civilisation are. Here we provided an integrated explanation of the necessary preconditions for science and civilisation. We have shown that some cultures (experimental groups) met the needed preconditions to develop contemporary science while other cultures (control groups) did not. And, among the preconditions, we have explained how our methodological toolbox – with *universal* methodological abilities of the mind and *adaptive* methods we develop collectively using our mind – is the main mechanism through which we have been directly able to develop vast bodies of knowledge and thus science and civilisation. New methods and tools we create better explain how we have developed science as the central driving force we are able to directly influence, and they are more observable and measurable in the knowledge we create, than other factors such as geography and culture. We can only understand the origins of science and civilisation, and thus what makes them possible and even conceivable, by understanding the evolutionary origins of our methodological toolbox that enables developing these achievements. This is the first paper to characterise science and its foundations in terms of the universal and adaptive methods that our species has used to acquire knowledge throughout history. We are a method-making species – indeed a hyper-method-making species. The success of our unique species in meeting our needs, solving problems and developing science and civilisation has been a success story of realising the power of our methodological abilities and developing new methods and tools using these abilities.

Observation, problem solving and experimentation are deeply embedded in how we have always and will always get by and acquire knowledge. Using our universal methodological toolbox we could develop science and civilisation, and by expanding our adaptive methodological toolbox with new methods and tools we eventually developed their more systematic form, contemporary science and contemporary society.

What are the conceptual implications here?

• We need to redescribe science as developed through the iterative process of using our mind's methodological abilities in increasingly systematic ways, throughout history, to develop new methods and tools and thus medical, agricultural and technological knowledge. Science and civilisation are built on cumulative methods and knowledge that we have developed, fine-tuned and passed along over time. We have driven the major revolutions throughout our history – the palaeolithic technological and agricultural revolutions and later the so-called scientific, industrial and digital revolutions – by using our methodological abilities in new ways and developing new methods and tools, i.e. through *methodological revolutions*.
• We need to abandon the common textbook view of the so-called scientific method as largely an invention of 17th century Europeans [57,60]. We need to no longer view science as a product of a few scholars, with something as complex and multidimensional as science not able to emerge at a single point in time.

What are the practical implications here? We can take important steps to advance science by:

• identifying and better understanding our evolved biological, cognitive and sensory constraints and biases, and improving our methods including tools to reduce them;
• combining multiple methods (within and across disciplines);
• identifying the constraints and assumptions of our methods and ways to reduce them;
• developing entirely new methods for solving new questions and problems we face.

Taking these steps is the main way we have been able to broaden our methodological scope and understanding of the world. Explaining science's past is of great importance for improving what drives and constrains its present development. Ultimately, advancing science requires that we expand our powerful methodological toolbox in new and innovative ways.

## Data availability

No primary data were used for the research in the article but rather secondary literature. Ethics declaration: Approval from an ethics committee and informed consent were not required for this article because no data for participants nor sensitive information were included.

## Author contribution statement

Alexander Krauss conceived and wrote the paper.

## Declaration of competing interest

The author declares that he has no known competing financial interests or personal relationships that could have appeared to influence the work reported in this paper.

## Footnotes

1 Another way researchers have attempted to address the question of the foundations of science is by adopting conceptual and theoretical methods [[18], [19], [20], [21], [22], [23]]. Philosophers and historians of science have argued that what is scientific and what is not depends on whether it is or is not logically verifiable [18], falsifiable [19,71], puzzle-solving and hypothesis-revising [72], guided by certain epistemic norms [22], advancing a research programme [23] and reflected in systematic knowledge [83]. Though, we focus here on scientific explanations.
